# ERK Activity in Immature Leukemic Cells Drives Clonal Selection during Induction Therapy for Acute Myeloid Leukemia

**DOI:** 10.1038/s41598-020-65061-6

**Published:** 2020-05-20

**Authors:** Michal Hayun, Maria Zaatra, Chen Itzkovich, Dvora Sahar, Dina Rosenberg, Margarita Filatova, Shimrit Ringelstein-Harlev, Hagit Baris, Nivin Moustafa-Hawash, Igal Louria-Hayon, Yishai Ofran

**Affiliations:** 1 The Clinical Research Institute at Rambam (CRIR), Rambam Health Care Campus, Haifa, Israel; 2 The Ruth and Bruce Rappaport Faculty of Medicine, Technion, Haifa, Israel; 3 Hematology Laboratory, Rambam Health Care Campus, Haifa, Israel; 4 Department of Hematology and Bone Marrow Transplantation, Rambam Health Care Campus, Haifa, Israel; 5 Genetics Institute, Rambam Health Care Campus, Haifa, Israel

**Keywords:** Acute myeloid leukaemia, Genetics research

## Abstract

Selection of resistant clones following intensive chemotherapy is a common obstacle for cure in many cancers, particularly in acute myeloid leukemia (AML). In AML, clone-specific sensitivity to chemotherapy varies even within the same patient. Multiple mutations and genetic aberrations are associated with clones surviving chemotherapy. The current study explored the role of activated signaling pathways in chemoresistance as a function of cell maturation, reflected by CD34 expression. *In-vitro*, Kasumi-1 leukemic cell line, sorted by CD34 expression, showed increased apoptosis only in the CD34^−^ subpopulation after exposure to cytosine arabinoside (Ara-C) or daunorubicin. The resistant CD34^+^ subset demonstrated higher expression of ERK1/2 and BCL-2 proteins than CD34^−^ cells. MEK1/2 inhibition elevated Ara-C ability to induce apoptosis in CD34^+^ cells, suggesting that MEK1/2-ERK1/2 is surviving signaling, which correlates to cell maturation levels and plays a role in chemoresistance. Deep sequencing of sorted CD34^+/−^ populations, both derived from the same patient samples, demonstrated various subclonal distribution of NPM1, DNMT3A and FLT3-ITD mutations. Interestingly, in these samples, p-ERK levels and apoptosis rates following chemotherapy exposure significantly differed between CD34^+/−^ populations. Hence, clones may be selected due to their ability to escape apoptosis rather than a direct effect of chemotherapy on a specific mutated clone.

## Introduction

Clonal selection is a major challenge for curing cancer patients treated with chemotherapy. Apparently, minor subclones distinguished from the main clone by various genetic and epigenetic aberrations are capable of surviving the effects of combination therapy that effectively eradicates the majority of cancer cells. These surviving subclones may cause a future relapse presenting with more aggressive features. Acute myeloid leukemia (AML) is a model of a molecularly heterogeneous malignant disease, known to exhibit several subclones at diagnosis. While the role of many mutations in leukemogenesis and disease progression is known, no mechanism linking clonal selection, occurring at the time of induction therapy to a specific mutation/variant combination has been identified in leukemia.

During induction chemotherapy including a combination of cytosine arabinoside (Ara-C) and daunorubicin (DNR) (“7 + 3” induction regimen), used as the first-line therapy, minor leukemic subclones survive and may further contribute to disease progression and/or relapse^1^. Although this clonal selection phenomenon occurs in a vast majority of AML patients^2–4^, selected subclones originating from different patients do not necessarily share specific mutation combinations. Recent studies have reported that combinations of the most common mutated genes, that are of prognostic value in AML, including Fms-like tyrosine kinase 3 - internal tandem duplication (*FLT3-ITD*), nucleophosmin 1 (*NPM1*) and DNA methyltransferase 3A *(DNMT3A)*, usually co-exist in different subclones within the same patient and are prone to cause chemo-refractoriness or early relapse^5–7^. Nevertheless, no specific subclone has been identified as the one likely to resist chemotherapy. Importantly, some pre-leukemic mutations, such as *DNMT3A*, are associated with age-related clonal hematopoiesis and may even be found in non-leukemic cells present in elderly without hematological disorders. At the same time, leukemic clones often carry a combination of several aberrations, including pre-leukemic mutations. Guryanova at el have excluded DNR efflux and metabolism or intracellular compartmentalization from the list of potential causes for chemoresistance in such cells^7^. These authors suggest that alterations in the DNA repair mechanism drive anthracycline resistance of leukemic cells. However, this finding does not explain the clonal selection observed in patients treated with non-anthracycline containing regimens or in those not carrying the *DNMT3A* R882 mutation.

Leukemic blasts are originally derived from a common primitive initiating cell which resides within the CD34^+^CD38^−^ cell fraction^8,9^. Impaired cell differentiation is one of the hallmarks of AML; still, leukemic blasts do undergo limited differentiation. Obviously, subclonal properties are determined by their genetics, epigenetics or post-translational modification. One may assume that each of the numerous existing subclones with its unique genetic mutation combination should be studied separately. Indeed, some resistance mechanisms, while being mutation-specific, are not associated with the differentiation stage of the mutated blasts. Several studies have shown that the expression level of CD34^10^, CD7^11^, CD25^12,13^ and CD56^14–16^, as measured in whole blast populations, is of prognostic value in AML. In addition, within the same patient, a different mutational profile of blast subpopulations is observed when blasts are sorted by markers of early differentiation, such as CD34/CD38^17–20^.

It is well recognized that even in most chemosensitive AML cases a significant number of leukemic cells survive the first cycle of chemotherapy^21^. Thus, the comparison of patient samples obtained at diagnosis and as early as Day 14 of induction, performed in the current study, cannot necessarily identify the cells which are resistant and may be the seeds of future relapse; however, this provides a unique opportunity to explore the process of clonal selection in real time.

The present study aimed to examine the chemosensitivity of CD34^+/−^ AML subclones during the first days of therapy, based on the expression of CD34, known to be the main marker distinguishing maturation stages of leukemic cells, and to explore differences in the ability of these subclones to escape apoptosis.

## Results

### Subpopulations of Kasumi-1 AML cell line exhibit different chemosensitivity properties

**Figure 1:**
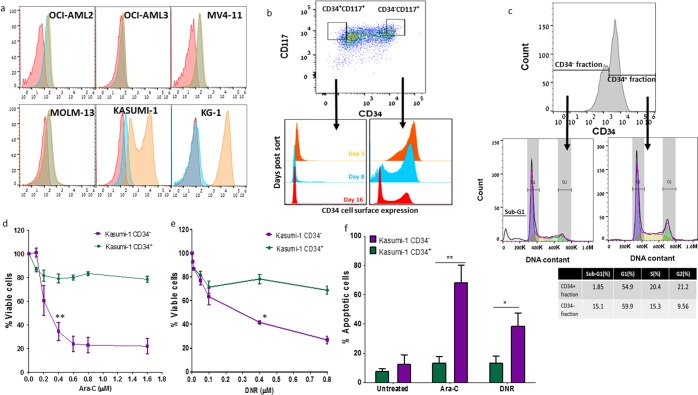
Kasumi-1 cell line sorted into CD34^+^ and CD34^-^ fractions exhibits unique growth behavior in culture and displays different chemosensitivity properties. (**a**) The CD34 expression in different AML cell lines (OCI-AML2, OCI-AML3, MV4-11, MOLM-13, Kasumi-1 and KG1) (orange curve) was evaluated by staining with anti-CD34 antibody. The results were compared to unstained cells (red curve) and matched isotype control (blue curve), using flow cytometry. (**b**) Kasumi-1 cells were sorted by fluorescence-activated cell sorting (FACS-Aria IIIu) according to CD34 and CD117 surface expression into CD34^+^ and CD34^-^ fractions. Each subpopulation was grown separately under the same conditions and the CD34 surface expression was examined in each fraction on days 3 (orange), 8 (blue) and 16 (red). (**c**) Cell cycle analysis of each gated fraction of Kasumi-1 cells (derived from CD34^+^ cells 13 days post-sorting) was performed according to their CD34 expression. The percentage of cells in CD34^+^/^-^ fractions at each cycle phase is presented. (**d**) Kasumi-1 CD34^+/-^ fractions were exposed to different concentrations of Ara-C (0-1.6 μM) or (**e**) DNR (0-0.8 μM) for 48 hours. Viable cells were measured by Fluorometer after additional 18-hour culture in the presence of alamarBlue reagent. (**f**) Percentage of apoptotic cells following incubation with Ara-C (0.4 μM) or DNR (0.2 μM) for 48 hours was determined using Annexin-V and PI staining. Results (d-f) are average ±SE of at least 3 independent experiments. *p < 0.05, **p < 0.01.

Six different human AML cell lines were examined for their CD34 cell surface expression level. Kasumi-1, derived from early myeloid stem cells and carrying the t(8:21)^22^ was found to express a wider spectrum of CD34 antigens compared to other examined cell lines (Fig. 1a) Therefore, Kasumi-1 cells were sorted into two fractions exhibiting either very high (CD34^+^) or very low (CD34^-^) expression of CD34. The fractions were then cultured separately. The t(8:21) was equally presented in both fractions. During culture, the CD34 expression level was monitored in each fraction on days 3, 8 and 16 post-sorting. While some of the sorted CD34^+^ cells differentiated into the CD34^-^ state, the sorted CD34^-^ subpopulation maintained its phenotype (Fig. 1b). Hence, the differentiation direction was from CD34^+^ to CD34^-^. The examination of cell cycle changes in each subset confirmed the difference between the fractions. CD34^+^ cells were more likely to exist in the S phase, whereas an increased number of CD34^-^ cells existed in the sub-G1 phase (Fig. 1c), meaning that two distinct subpopulations co-existed within the same cell line.

In an attempt to evaluate whether along with phenotypic differences the CD34^+/-^ fractions displayed different functional abilities, we examined the sensitivity of Kasumi-1 CD34^+/-^ subpopulations to increasing concentrations of Ara-C (0–1.6 μM) or DNR (0-0.8 μM) following incubation with each drug alone. Kasumi-1 CD34^+^ cells appeared to be resistant to Ara-C at all tested concentrations, while CD34^-^ cells were sensitive to the concentrations higher than 0.4 μM (Fig. 1d). Following DNR exposure, at doses higher than 0.4 μM, a similar pattern was observed (Fig. 1e). The apoptosis assay confirmed these results: the apoptosis rate in the CD34^-^ subpopulation was 4-fold and 2.5-fold higher than in the CD34^+^ subpopulation following exposure to Ara-C and DNR, respectively (Fig. 1f).

### Involvement of MEK/ERK signaling in chemosensitivity of CD34^+/-^ subsets

**Figure 2:**
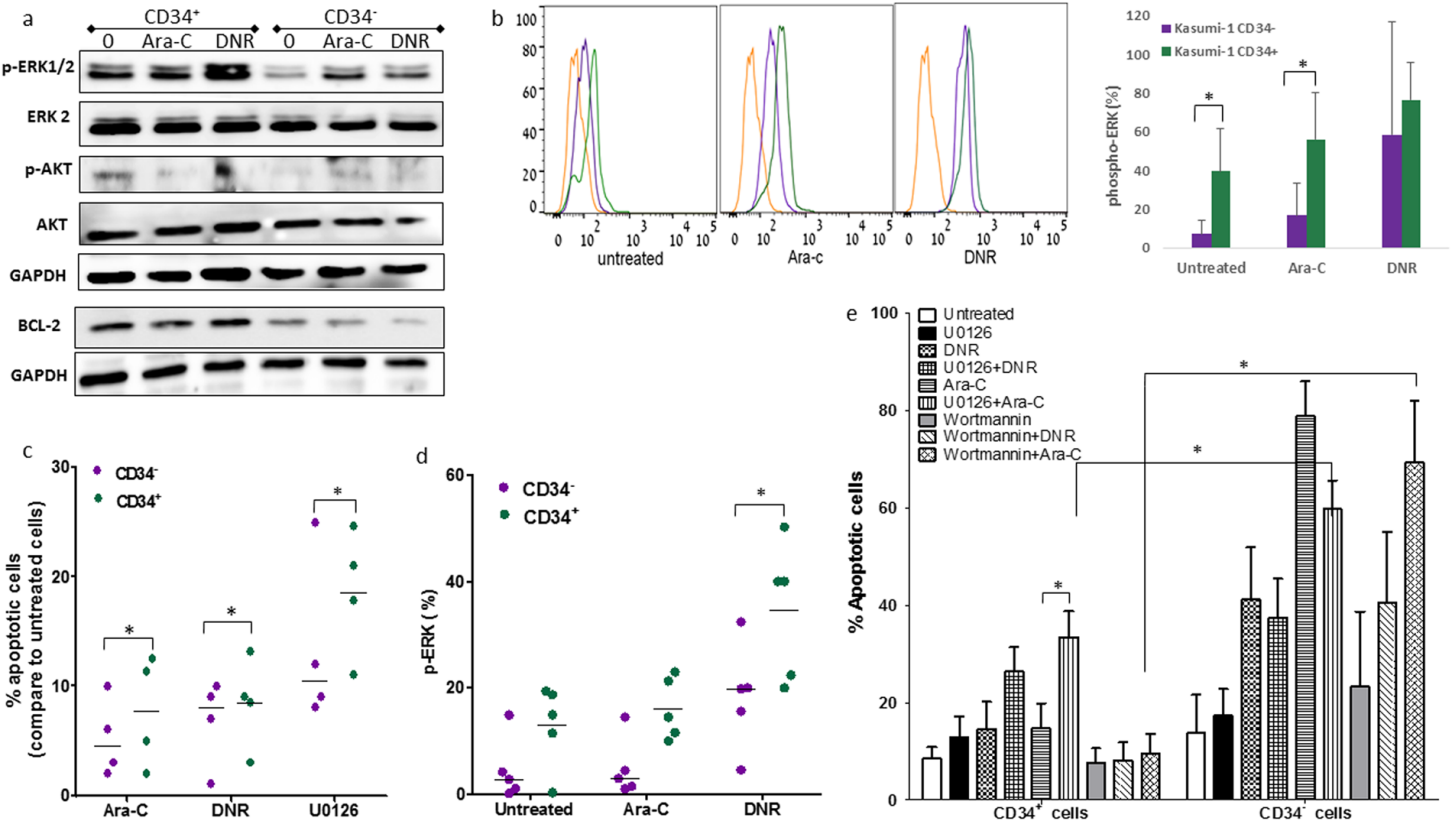
Involvement of MEK1/2-ERK1/2 signaling pathway in chemosensitivity of Kasumi-1 CD34^+/-^ subsets. (**a**) Kasumi-1 CD34^+/-^ cell fractions were treated for 1 hour with Ara-C (0.4 μM) or DNR (0.2 μM). Cell lysates were extracted and equal amounts of total protein were subjected to SDS-PAGE. Western blot assay was performed with antibodies against p-ERK1/2, ERK2, p-AKT, AKT, BCL-2 and GAPDH as loading control. Representative of 3 biological replicates. Samples were derived from the same experiment; blots were processed in parallel. The full-length gels and blots are included in the Supplementary Information file. (**b**) FACS representative analysis of p-ERK1/2 (pT202/pY204) expression in Kasumi-1 CD34^+/-^ subsets at baseline and following 1-hour exposure to Ara-C or DNR was conducted. Average ±SE of at least 3 experiments in each cell fraction is summarized (unstained cells: yellow; CD34^-^ subset: purple; CD34^+^ subset: green). (**c**) Expression levels of p-ERK1/2 (pT202/pY204) in CD34^+/-^ subsets in BM samples of five patients harboring t(8:21) at baseline and following 1-hour exposure to Ara-C (0.4 μM) or DNR (0.2 μM) were assessed by FACS. *p < 0.05. (**d**) Apoptosis induction by Ara-C (0.4 μM), DNR (0.2 μM) or U0126 (30 μM) in four primary samples of patients harboring t(8:21) was determined after 24 hours using Annexin-V and PI staining. The results are presented in comparison to spontaneous apoptosis in culture. (**e**) Either the MEK1/2 inhibitor U0126 (30 μM), or the PI3-k inhibitor Wortmannin (3 μM) was added to Kasumi-1 cells 2 hours prior to Ara-C (0.4 μM) or DNR (0.2 μM) exposure. Apoptosis was determined after 48 hours using Annexin-V and PI staining. Results are average ±SD of at least 3 independent experiments. *p < 0.05.

The Mitogen-activated protein kinase kinase 1/ extracellular signal-regulated kinases 1/2 (MEK/ERK) pathway is known to be survival signaling of cancer cells. We examined potential differences in the activation of MEK/ERK signaling between CD34^+^ and CD34^-^ subsets of Kasumi-1 cells as well as in the BM specimens obtained from five AML patients carrying t(8:21) upon Ara-C or DNR exposure. In the untreated cells, the baseline p-ERK1/2 activity was significantly higher in the CD34^+^ subset of Kasumi-1 cells compared to CD34^-^ subset, as measured both by western blot (Fig. 2a) and phosflow assays (an average of 38.2% in CD34^+^ vs. 5.2% in CD34^-^ subpopulations; Fig. 2b). Upon exposure to chemotherapeutic drugs, ERK phosphorylation increased in both fractions (Fig. 2a,b), but was significantly higher in the CD34^+^ subpopulation. This was observed both in Kasumi-1 cells (Fig. 2a,b) and patient specimens (Fig. 2c). In CD34^+^ blasts derived from patients with t(8:21), MEK1/2 inhibition using a selective inhibitor U0126 resulted in marked apoptosis, which was even higher than that observed with Ara-C or DNR treatments (Fig. 2d). The use of U0126 alone did not cause apoptosis in either CD34^+^ or CD34^-^ Kasumi-1 subpopulation (Fig. 2e). However, incubation of the cells with U0126 before chemotherapy exposure, sensitized them to Ara-C introduction, which resulted in a significant increase in apoptosis of CD34^+^ cells (p = 0.03). A similar, albeit non-significant, pattern was observed following DNR exposure. The strong effect of Ara-C and DNR in inducing apoptosis in the CD34^-^ cells mainly resulted from their low expression of both p-ERK and the anti-apoptotic BCL-2 protein (Fig. 2a), which probably overwhelmed the inhibitory effect of U0126. Additionally, p-AKT did not seem to be involved in chemosensitivity of Kasumi-1 CD34^+/-^ subsets, since incubation of CD34^+/-^ subsets with the phosphoinositide 3-kinase (PI3-k)/AKT inhibitor Wortmannin did not either enhance or abrogate the apoptosis induced by Ara-C/DNR in any Kasumi-1 cell subset (Fig. 2e).

**Figure 3:**
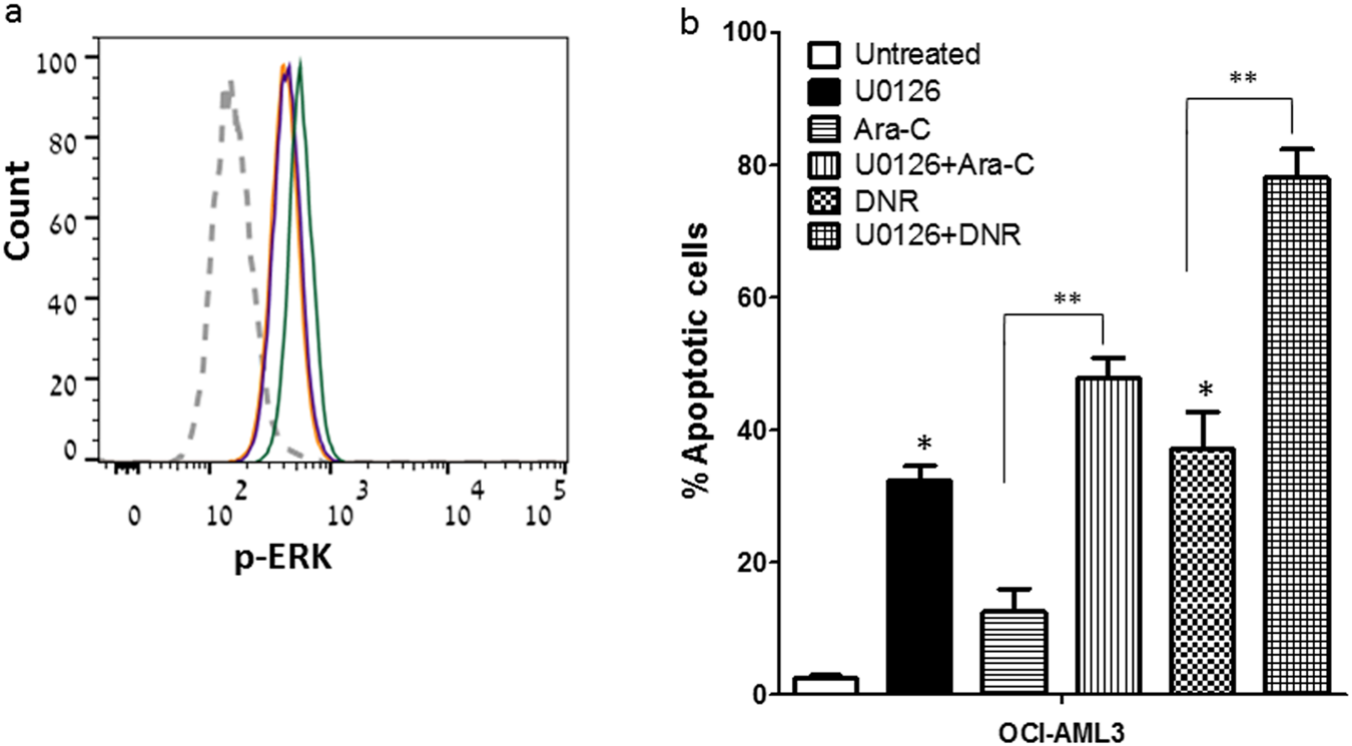
Involvement of ERK activity in OCI-AML3 cell survival. (**a**) Expression levels of p-ERK1/2 (pT202/pY204) as measured by FACS analysis in OCI-AML3 cell line (unstained cells: grey; untreated: yellow; Ara-C: purple; DNR: green). (**b**) OCI-AML3 cells were exposed to U0126 (30 μM) 2 hours prior to Ara-C (6.4 μM) or DNR (0.2 μM) exposure. Apoptosis was determined after 48 hours using Annexin-V and PI staining. Results are average ± SD of 3 independent experiments. *p < 0.005 increase vs. untreated, **p < 0.01 increase vs or U0126 + Ara-C or U0126 + DNR.

Another AML cell line, OCI-AML3, has been used to further explore the effects of ERK on AML cell survival. This cell line has been shown to be even more resistant to chemotherapy than Kasumi-1 cell line (Ara-C IC_50_: 6.4 µM versus 0.4 µM, respectively). While OCI-AML3 has been found to exhibit high ERK activity (Fig. 3a), its inhibition with U0126 alone has resulted in an up to 30% increase in apoptosis of these cells, which has been further enhanced with the addition of either Ara-C or DNR (up to ~50 and ~80%, respectively) (Fig. 3b).

### Changes in CD34^+/-^ blast subpopulation composition during therapy

To screen the blast cell dynamics at different stages of differentiation during induction therapy for AML, samples of sixty consecutive patients receiving the “7 + 3” regimen were tested (Table 1).

**Table 1:** Patient characteristics.

	Percentage	Median	Range
Gender (male)	53.3		
Age (years)		55.5	19–74
WBC at presentation (k/µl)		8.3	0.8–397.8
**ELN risk**			
Standard	21.6		
Intermediate	46.6		
Poor	31.6		
Secondary AML	11.6		
**Mutations**			
NPM1	20		
FLT3-ITD	13.3		
NPM1and FLT3-ITD	25		

The percentage of CD34 expressing blasts out of all the bulk cells within the blast window was recorded at diagnosis (Day-1, pre-treatment) and on Day-14 (post-treatment) of induction therapy. When all the patients were analyzed together, good responders, defined by the presence of <10% of blasts in the Day-14 BM, were found to exhibit a significant change in their CD34^+^ blast fraction by Day-14. The fold change in the number of CD34^+^ cells was calculated as the ratio of the higher/lower percentage values of CD34 expressing blasts on Days 1 and 14 in each patient. When the CD34^+^ fraction increased from day 1 to day 14, the value of the fold change was considered negative.

**Figure 4:**
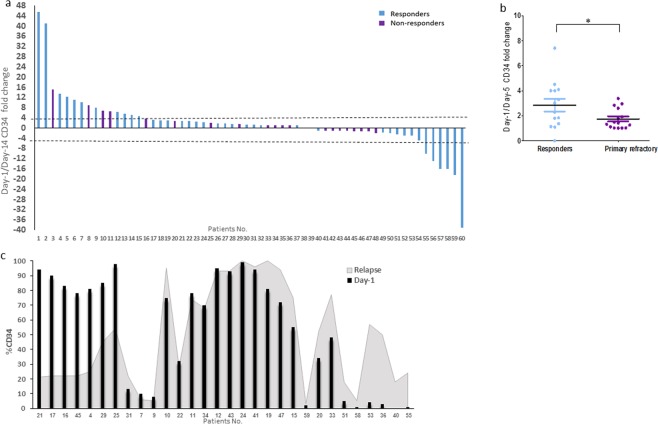
Changes in CD34 expression levels on BM blasts at diagnosis (Day-1) and after induction therapy (Day-14) in correlation with responses to therapy. The CD34 expression level on Day-1, Day-5, Day-14 and relapsed BM blasts, derived from AML patients receiving the “7 + 3” protocol, was measured by FACS. (**a**) A fold change in the CD34 expression between Day-1 and Day-14 (water fall plot, n = 60) or (**b**) between Day-1 and Day-5 BM blasts (p < 0.05, n = 29) presented with regard to patient response to treatment. Results are presented as mean ± SE. (**c**) The expression of CD34 cell fraction percentage on blasts derived from diagnosis and relapse (n = 30).

The absolute value of the calculated “fold change” was significantly higher among good responders (mean 8.252 ± 1.761; n = 39), compared to primary refractory patients (mean 2.936 ± 0.782; n = 21) (Fig. 4a). The change in the CD34 cell fraction was minimal and significantly lower in non-responders compared to responders [17/21(81%) and 21/39 (53.8%), respectively; p = 0.0077]. A similar fold change pattern in the CD34 expression was demonstrated when BM blasts were examined as early as Day-5 of induction (Fig. 4b). It is of interest that among responders the CD34^+^ cell fraction was either increased or decreased following induction in similar portions of patients. In non-responders there was no selection for the CD34^+^ subset. Notably, no association was found between the appearance of a specific mutation and the ability to respond to treatment.

Thirty out of sixty patients (50%) relapsed (Fig. 4c). Twenty/30 relapsed patients (66.6%) exhibited a large CD34^+^ cell fraction at diagnosis and in most of them this fraction was also increased in relapse.

### CD34^+/−^ subpopulations differ in their subclonal composition

**Figure 5:**
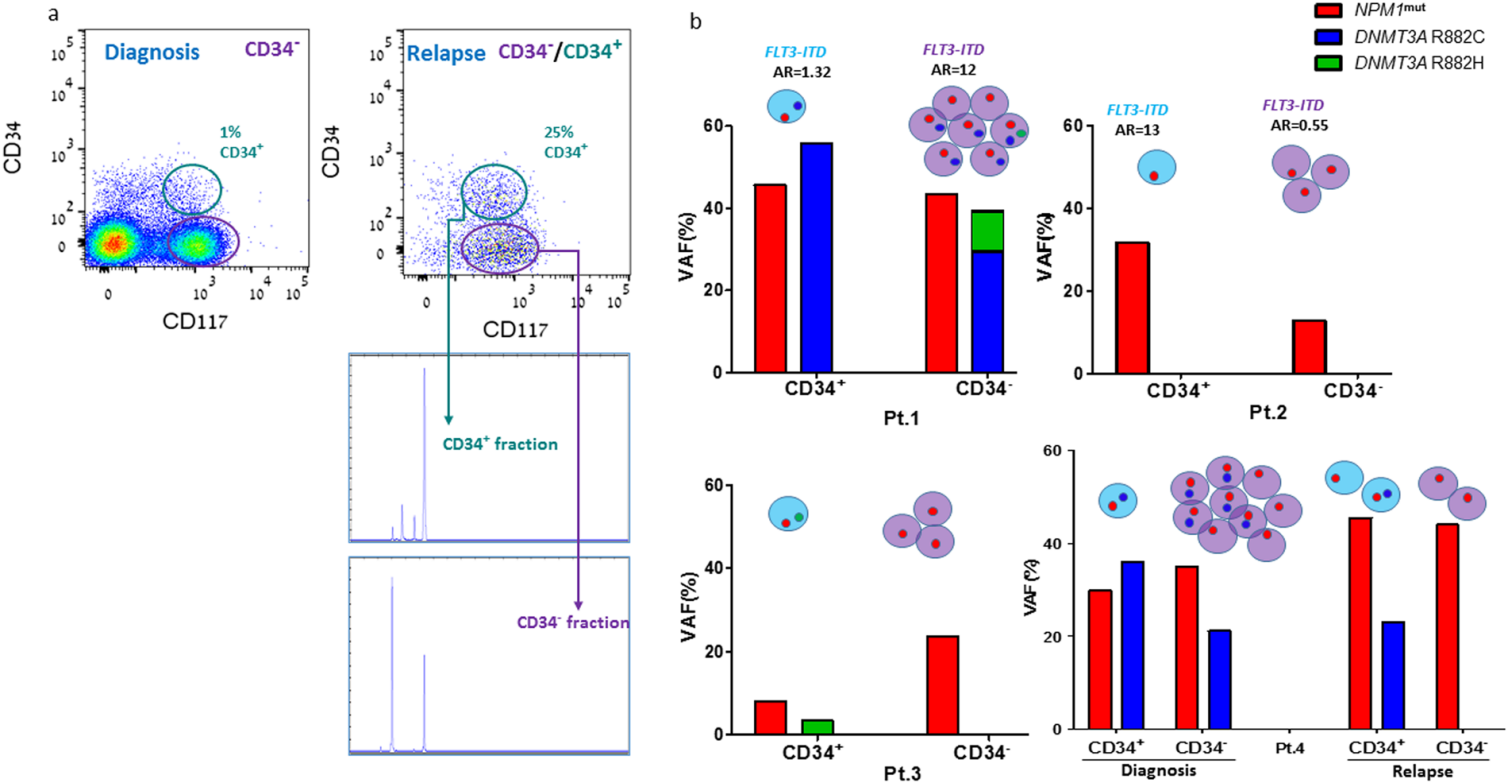
Mutation variants identified in CD34^+^ and CD34^-^ AML blast subsets and their allelic frequency. (**a**) FACS analysis shows blast cells derived from the same AML patient at diagnosis and relapse, and sorted to CD34^+^CD117^+^ and CD34^-^CD117^+^ fractions. DNA was extracted from each fraction and PCR products were examined by genetic analyzer for *FLT3-IDT* mutation. Mutant *FLT3-ITD* (right column) and wt-*FLT3* (left column) alleles are shown in each CD34 compartment. (**b**) BM blasts from 4 AML patients were sorted into CD34^+^CD117^+^ and CD34^-^CD117^+^ fractions. Genomic DNA was extracted from each subset; targeted sequencing conducted using PGM Ion Torrent, qualified by coverage of ~2000 reads, was performed for: *NPM1 and DNMT3A* mutations. All the 4 patients carried *NPM1*^*mut*^ (TCTG insertion) and *FLT3-ITD* mutations. In the *DNMT3A* hotspot region, two variants were identified: R882H and R882C. The allelic frequency of the variants in CD34^+^ and CD34^-^ subpopulations is presented for each patient. The calculated allelic ratio (AR) for *FLT3-ITD* mutation (detected using the Genescan method) in each fraction is presented, when available. The circles, denoting cells, represent the ratio between the numbers of blast cells in each fraction.

Nine *FLT3-ITD* positive AML patients were tested and their cells in the blast window were sorted into CD34^+^CD117^+^ and CD34^-^CD117^+^ subsets. The allelic ratio (AR) of *FLT3-ITD* in CD34^+/-^ subpopulations was measured separately for each patient (Suppl. Figure 2). AR differences of ≥1.25 were unlikely to reflect technical test issues and were considered biologically significant. Such differences between CD34^+/-^ fractions were detected in 7/9 patients (88%; Pts. C-I, Table 2). The loss of the *FLT3*^wt^ allele and an expansion in the CD34^+^ cell subset were observed at relapse in a representative patient sample (pt. I, Table 2; Fig. 5a).

**Table 2:** The allelic ratio of *FLT3-ITD* mutation in each blast subset. AR changes were calculated by dividing the high AR by low AR of each CD34+/- subpopulation for each patient.

Pt. No.	CD34^−^	CD34^+^	AR changes
A	0.98	1.01	1.03
B	1.1	1.03	−1.07
C	0.98	0.71	−1.38
D	1.24	1.73	1.40
E	0.89	1.38	1.55
F	0.99	0.12	−8.25
G	12	1.32	−9.09
H	0.63	0.05	−12.60
I	0.55	13	23.64

To evaluate the diversity of common AML-related mutations (*FLT3-ITD*, *NPM1, DNMT3A*,) in CD34^+/-^ subpopulations within the same patient, 4 patients co-expressing these 3 mutations were selected for assessment (Pts.1-4, Fig. 5b). Banked BM specimens, cryopreserved prior to therapy initiation, were sorted according to the CD34 expression. The combination of the above-mentioned mutations in the same cell was suggested to be associated with chemoresistance^7^ and if so, one would expect the subclones carrying all the three mutations to expand particularly at relapse.

In patient #1 (pt. G, Table 2), a 47-year old male, *NPM1*^*mut*^ and not *DNMT3A*^*mut*^ (R882C) or *FLT3-ITD* mutated allele was equally distributed among CD34^+^ and CD34^-^ cells (allelic frequency of 45.6% vs 43.5% for *NPM1* and 55.8% vs 29.4% for *DNMT3A;* AR of 1.32 vs 12 for *FLT3-ITD*, respectively). Remarkably, cells carrying the *DNMT3A* R882H variant were also found in this patient, albeit within the CD34^-^ subpopulation only. In patient #2 (pt. I, Table 2), a 33-year old male, 63% of CD34^+^ cells compared to 25% of CD34^-^ cells were *NPM1*^*mut*^. The *FLT3-ITD* AR for CD34^+/-^ subpopulations was 13 vs 0.55. In patient #3 (a 32-year old female, relapse sample, AML transformed from MDS), 47% of blast cells within the CD34^-^ fraction and only 14% of CD34^+^ cells expressed *NPM1*^*mut*^ (allelic frequency of 23.7% vs 7%). *DNMT3A* mutation (R882H) was detected solely in the CD34^+^ fraction, with an allelic frequency of 6.8%. In patient #4 (a 71-year old male), samples from diagnosis and relapse were examined. At diagnosis, the *NPM1*^*mut*^ subpopulation was similarly distributed among CD34^+/-^ fractions (70% in CD34^+^ and 60% in CD34^-^), but *DNMT3A*^*mut*^ (R882C) was more frequent in the CD34^+^ subpopulation (36% vs 21%). At relapse, while *NPM1*^*mut*^ alleles maintained their frequency in both CD34^+/-^ fractions, cells with the *DNMT3A*^*mut*^ allele were present within the CD34^+^ fraction only. This result pointed to unequal distribution of mutations among CD34^+^ and CD34^-^ fractions within the same patient.

### CD34^+/-^ subpopulations differ in ERK activity and cell apoptosis induced by chemotherapy

**Figure 6:**
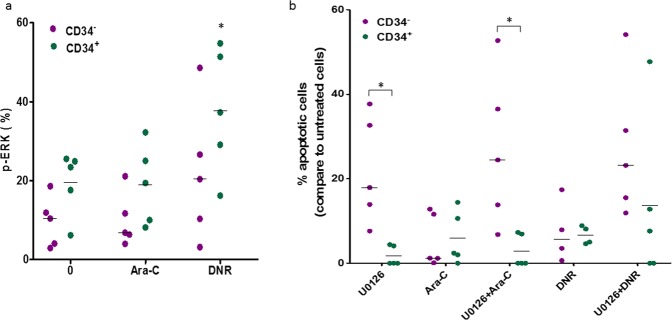
Involvement of MEK1/2-ERK1/2 signaling pathway in chemosensitivity of CD34^+/-^ subsets from BM blasts harboring *FLT3-ITD*. FACS analysis of CD34^+^CD117^+^ or CD34^-^CD117^+^ fractions derived from BM of five patients harboring *FLT3-ITD* presented in Table 2. (**a**) p-ERK1/2 (pT202/pY204) expression levels in CD34^+^CD117^+^ or CD34^-^CD117^+^ subsets following 1-hour exposure to Ara-C (0.4 μM) or DNR (0.2 μM). *p < 0.05 increase vs. untreated or Ara-C-treated in CD34^+^ fraction. (**b**) Apoptosis induction by Ara-C (0.4 μM), DNR (0.2 μM) or U0126 (30 μM) after 24-hour exposure was determined using Annexin-V staining. The results are presented in comparison to spontaneous apoptosis in culture. *p < 0.05.

MEK/ERK signaling activity was further examined in 5/9 above-mentioned patients (A, C, E, G, H; Table 2) whose samples were available for the analysis. Gating strategy for CD34^+/-^ analysis was identical to the protocol used for the cell separation (described above). p-ERK expression levels significantly increased in response to DNR exposure in the CD34^+^ but not in the CD34^-^ fraction (Fig. 6a). In both CD34 fractions, similar apoptosis rates were observed following 24 hours of incubation with Ara-C or DNR. Interestingly, inhibition of MEK1/2 resulted in a marked increase in the apoptosis rate within the CD34^-^ subset only (Fig. 6b). These results in primary patients’ specimens are in accordance with our observation in Kasumi-1 cells. Notably, differences in the ERK activity and cell survival following chemotherapy exposure and unbalanced segregation of subclones co-exist within CD34^+/-^ subsets derived from patients.

## Discussion

Understanding the underlying mechanism that drives chemotherapy-induced subclonal selection may have an important bearing on the improvement of AML treatment. At early maturation stages, cells, regardless of their genotype, are less vulnerable to chemotherapy due to intense survival signaling. Upon exposure to chemotherapy, a residual population of immature cells enriched with subclones of limited maturation capacity, survives and ultimately becomes dominant. Our findings suggest that it is the differentiation level of a subclone that could determine its survival under the pressure of intensive induction chemotherapy.

Using the human Kasumi-1 AML cell line, we demonstrated that the sorted subpopulations (CD34^+^/^-^), despite sharing the same genetic background, displayed differences in chemosensitivity. Notably, in our study, this cell line proved to be an appropriate model to sort cells according to their differentiation stage. In Kasumi-1, the initiating cells appeared to be CD34^+^; thus, this self-renewing culture could only be produced by this subpopulation. Pedranzini and colleagues reported that Kasumi-1 cells kept in continuous culture, showed a dramatic decrease in the CD34^+^ subpopulation, leading to depletion of the CD34^+^CD38^-^ compartment^23^.

In normal hematopoiesis, ERK signaling is suggested to be involved in the regulation and maintenance of early myeloid commitment of hematopoietic stem cells (HSCs)^24,25^ and is required for granulocytopoiesis and monocytopoiesis^26^. Moreover, ERK activation is essential for final maturation of erythrocytes and monocytes, whereas initial ERK activity is required for optimal expansion of progenitors during myelopoiesis^27^.

Based on these data, we explored ERK signaling in CD34^+^/^-^ subpopulations. Our findings demonstrated differences in the regulation of MEK1/2-ERK1/2 signaling pathway between mature and immature subpopulations, both at the baseline level and following *in-vitro* DNR or Ara-C exposure. Notably, down-regulation in the anti-apoptotic protein BCL-2 level was observed only in the CD34^-^ fraction. These results of ours are in line with the data by van Stijn *et al*., who reported a correlation between resistance to spontaneous apoptosis and BCL-2 expression in CD34 subsets, which was more prominent in the CD34^+^ than CD34- fraction^28^. However, that latter study did not use chemotherapy to explore such differences between the fractions and did not examine MEK/ERK involvement.

In untreated Kasumi-1 cells (as well as in AML blasts expressing CD34), the baseline p-ERK level was higher in the CD34^+^ than in the CD34^-^ subpopulation. Thus, one may assume that the elevated p-ERK levels could protect these cells from the lethal effects of oxidative stress induced by DNR and Ara-C. Additionally, mitogen-activated protein kinase (MAPK) pathways were reported to contribute to the poor AML prognosis and development of refractoriness to Ara-C^29–31^.

In the OCI-AML3 cells, exhibiting high baseline ERK activity leading to their resistance to Ara-C/DNR, a combination of the MEK1/2 inhibitor U0126 with these chemotherapeutic agents has significantly enhanced AML cell apoptosis. This strategy might have clinical implication in AML patients.

Resistance of Kasumi-1 CD34^+^ cells to chemotherapy appeared to be partially abrogated by ERK inhibition, while the addition of U0126 to CD34^-^ cells did not significantly affect their response to chemotherapy. These findings suggested that MEK/ERK pathway could be the survival signaling mediating chemoresistance mainly in the CD34^+^ subset. Our results demonstrating increased ERK phosphorylation at Thr202 and Tyr204 (a marker of MEK activation) residues in Kasumi-1 cells, without AKT activation, are supported by earlier findings of higher ERK activity in Kasumi-1 cells compared to other AML cells, with no significant phosphorylation of Ser473 of AKT^32^.

We have shown that the activity of ERK1/2 kinases in AML is linked to the cell differentiation stage, irrespective of genetic alterations, i.e., the pattern of kinase pathway activity differs in CD34^+/-^ populations. In primary leukemic cells derived from 5 *FLT3-ITD* patients, CD34^+/-^ subsets differ in their p-ERK expression and ability to survive *in-vitro* in response to chemotherapy. They also have different genetic composition. We assume that these differences may represent inherent features shared by all hematopoietic cells at a certain level of differentiation and not necessarily disease-related properties. Since AML subclones differ in their differentiation capacity, under chemotherapy pressure, leukemic cells at their early differentiation stages are selected via resistance mechanisms involving cell survival signaling pathways.

Guryanova *et al*. have recently demonstrated that in AML, anthracycline resistance is related to a DNA repair mechanism in *DNMT3A* R882 mutated cells^7^. This may explain clonal selection in a specific subpopulation of AML patients harboring this mutation. However, since clonal selection is common in AML, regardless of a specific genotype, we assume that the existence of a shared subclonal phenotype is associated with chemoresistance across different mutation combinations.

Remarkably, in the current study, only good responders demonstrated a significant change (either increase or decrease) in the CD34 expression between the blasts derived at diagnosis and later during therapy. This may imply that such phenomenon is common among AML patients and not restricted to a specific genotype. The lack of changes in the CD34 expression pattern in patients who failed induction could probably be explained by chemoresistance of the major clone in such primary refractory cases. Dynamic changes in the percentage of CD34 cells within a single patient during induction could represent accumulation of surviving resistant subclones, differing in their differentiation capacity from the main sensitive subclone. The described change in the composition of CD34^+/-^ subpopulations may help predicting patient’s response early during induction therapy. It is of interest that in relapsed patients the CD34^+^ cell subpopulation appears to be dominant both at diagnosis and relapse, while no significant differences in the CD34 population size have been revealed at these two time points. However, one should bear in mind that no definitive conclusion can be made as to whether CD34^+^ or CD34^-^ is the immature blast population that could be associated with relapse.

This study has several limitations. Our finding of the CD34^+^ subset as an immature and resistant subpopulation may be restricted to Kasumi-1 and other cell lines expressing CD34^+^ as the bulk cell marker. However, while we originally screened six leukemia cell lines for CD34 expression, Kasumi-1 was the only cell line that we ultimately decided to use as a model for chemosensitivity evaluation, since the differentiation stage of each subpopulation could be separately examined during culture. Sorting of patient-derived primary leukemic cells by their CD34 expression demonstrated that even within a homogenous group of patients (FLT3-ITD mutated or combined NPM1, FLT3, DNMT3A mutated) two cell subpopulations (CD34^+^/CD34^-^) differing in their genetic subclonal combination could be identified. Thus, an experiment exploring the effect of differentiation stage only on chemosensitivity of patients’ leukemia cells will always be subject to bias due to the differences in the genetic background of the sorted cells. Monitoring ERK activity at different time points in the same patient is subject to bias, since due to the clonal selection CD34^+^/CD34^-^ subpopulations have a different genetic background.

Our results have proven the concept that within the same patient, subclones have diverse differentiation capacity. Hence, CD34^+/-^ subpopulations have different mutational compositions and their epigenetic and signal transduction properties should be studied separately. Larger studies are required to determine if a specific combination of mutations creates subclones that have identical differentiation capacities and chemosensitivity in different patients.

In conclusion, in addition to the well-recognized genetic analysis, the current study has demonstrated the importance of the cell maturation stage evaluation for predicting response to chemotherapy. Changes in the size of CD34^+/-^ subpopulations during induction therapy could early predict patient’s response to treatment. Our findings show that ERK activity enhances survival and chemoresistance of CD34^+^ cells, thus indirectly contributing to clonal selection. Further studies are required to best identify patients in whom clinical use of ERK/MEK inhibition could be recommended.

## Materials and Methods

### Collection of AML blasts

Whole blood and bone marrow (BM) aspirates were collected from AML patients presenting to the Rambam Department of Hematology. All patients gave informed consent. All methods were performed in accordance with the relevant guidelines and regulations. Mononuclear cells were separated by centrifugation over a layer of LymphoprepTM (Axis-Shield PoC AS, Oslo, Norway) and then stored in freezing medium [fetal bovine serum (FBS) with 10% DMSO] in a liquid nitrogen tank.

### Cell culture

The human myeloid leukemia cell line Kasumi-1 was purchased from ATCC, maintained in the RPMI-1640 medium, supplemented with 20% FBS, 2 mM L-glutamine, and 1% penicillin-streptomycin (Biological Industries, Kibbutz Beit-Haemek, Israel) and incubated at 37 °C in 5% CO_2_. KG-1 cells were purchased from ATCC; OCI-AML2 and OCI-AML3 cell lines were obtained from the laboratory of Prof. Minden (Princess Margaret Cancer Centre, Toronto, Ontario, Canada). All these cell lines were maintained in Iscove’s Modified Dulbecco’s medium supplemented with 20% FBS. MOLM-13 cells were purchased from DSMZ and maintained in RPMI-1640 medium with 10% FBS.

The expression of CD34 in each cell line was measured by flow cytometry: 1×10^6^ cells were incubated with the FcR blocking reagent for 10 min at 4 °C and then either B.V421 anti-IgG2β isotype control (clone MOPC-173, BD Biosciences, San Jose, CA) or B.V421 anti-CD34 (clone 561, Biolegend, San Diego, CA) was added for 15 min at the room temperature (RT) in darkness. Cells were then washed with PBS and analyzed.

### Reagents

Ara-C (cat. C1768), DNR (cat. D8809) and the PI3-k inhibitor Wortmannin were purchased from Sigma (St. Louis, MO, USA). The selective MEK1/2 inhibitor U0126 was purchased from Cell Signaling Technology, Inc (Danvers, MA).

### Cell sorting

AML blasts selected by P.B anti-CD45 (clone 2D1, Biolegend) vs. SSC were stained with anti-CD34 (PE or FITC, clone 8G12, BD Biosciences) and APC-anti-CD117 (clone 104D2, Biolegend). Kasumi-1 cells and AML blasts were sorted by fluorescence-activated cell sorting (FACS-Aria IIIu, BD Biosciences) into CD34^+^CD117^+^ and CD34^-^CD117^+^ subpopulations. CD34^-^ and CD34^+^ cells were defined according to the strength of CD34 signal relative to the corresponding controls. Only cells displaying very high and very low CD34 staining were sorted out to ensure significant phenotypic difference between populations. Immediately after sorting, cells were cultured; DNA was extracted or cells were frozen as pellets.

### Cell viability assay

Kasumi-1 or OCI-AML3 CD34^+/-^ subsets were seeded (4×10^4^ cells/200 μl) in triplicates in the presence of Ara-C (0–1.6 μM and 0–12.8 μM, respectively) or DNR (0–0.8 μM for both cell lines). After 48 hours, 20 μl/well of alamarBlue reagent (Bio-Rad Laboratories, Inc.) were added and after additional 18 hours, viable cells were measured using fluorometer (FLUOstar Galaxy, BMG Labtechnologies, Germany). The mean number of viable cells at varying concentrations of the drugs was normalized to the number of untreated (no-drug) cells and percentages were calculated.

### Cell cycle distribution

Kasumi-1 cells were stained with B.V421 anti-CD34 (Biolegend) for 15 min, at RT, in darkness and then ethanol-fixed overnight at −30 °C. Fixed cells were washed with PBS, stained with propidium iodide (PI), treated with RNase A, and analyzed on the Attune NxT Flow Cytometer (Thermo Fisher Scientific Inc).

### Apoptosis assay

The ability of Ara-C, DNR, U0126 or Wortmannin to induce apoptosis was measured using the APC-Annexin-V Apoptosis Detection Kit with PI (Biolegend). Apoptotic cells were defined as a sum of early and late apoptosis events by flow cytometry. The analysis was conducted using the FlowJo software version 7.6.4.

### Phosflow method to detect ERK activation

Cell populations of Kasumi-1, OCI-AML3 or frozen BM specimens from patients diagnosed with t(8:21) AML were seeded (0.5 × 10^6^/ml) and rested in the incubator at 37 °C for 2 hours. Cells were then stimulated with Ara-C or DNR for 1 hour, fixed by Cytofix Buffer (BD Cytofix™ Fixation Buffer, BD Biosciences) and incubated at 37 °C for additional 10 minutes. Cells were further centrifuged at 400 g for 8 minutes, permeabilized by adding Perm Buffer (BD Phosflow™ Perm Buffer III) and incubated for 30 minutes on ice. Then, cells were washed twice with PBS containing 2% FBS, and Alexa- Fluro 488 anti-ERK1/2 (pT202/pY204) (clone 20 A, BD Bioscience); BV421 anti-CD34 (Biolegend) or APC-CD117 (Biolegend) antibodies were added for 30 minutes at RT in darkness. Finally, cells were washed, suspended in PBS and analyzed by flow cytometry.

### Western blot analysis

Cell extracts from sorted Kasumi-1 CD34^+/-^ fractions were prepared by cell suspending in ice-cold lysis buffer, containing 50 mM Tris (pH 7.4), 150 mM NaCl, 1 mM ethylenediaminetetraacetic acid, 10% glycerol, 1% Triton X, 1 mM sodium vanadate, 1 mM phenylmethylsulfonyl fluoride, 5 μg/ml aprotonin and 5 μg/ml leupeptin. Pellets were boiled for 10 min, electrophoresed in 10–12% sodium dodecyl sulfate – polyacrylamide gel, transferred to 0.45 μm PVDF membrane and immersed for 1 hour in a blocking solution (0.5% Tween-20, 5% BSA or milk). The membranes were incubated overnight with primary antibodies: mouse-anti-p44/p42 (pT202/pY204; cat#9101 S), rabbit-anti-p42 (cat#9108 S), rabbit-anti-pAKT (ser473; cat# 4060 S), rabbit-anti-AKT (1,2,3; cat#9272) all from Cell Signaling Inc., hamster-anti-BCL-2 (BD pharmingen; cat#51-1513GR) and mouse-anti-GAPDH (Abcam; cat#ab9484). The blots were washed three times with TBST buffer [10 mM Tris (pH 7.4), 100 mM NaCl 0.5%-Tween-20], incubated for 1 hour with secondary antibodies conjugated to horseradish peroxidase and re-washed. Blots were developed using the WesternBright ECL HRP substrate (Advansta Inc, San Jose, CA) in ImageQuant LAS 4000 (GE Healthcare).

### Immunophenotype and genetic data of AML patients

BM samples from consecutive 32 males and 28 females, aged 19-74 years, were collected on Day-1, Day-5 or Day-14 of the “7 + 3” induction protocol or at relapse. Leukemic blasts were identified and counted by FACS according to SSC vs. CD45 blast window and stained with FITC-anti-CD34 antibody (BD Biosciences) (Suppl. Figure 1). In 17 patients whose percentage of CD34 blasts was “0” (on Day-1, Day-5 or Day-14) the percentage value was changed to “1”. A “fold change” in the CD34 expression was calculated by dividing the highest CD34 percentage on BM blasts on Day-1 vs. Day-5 or Day-1 vs. Day-14 by the lowest CD34 percentage value found on these days.

### Polymerase chain reaction (PCR) amplification

Genomic DNA was extracted from each of the sorted fractions using the Exgene Genomic DNA micro kit (GeneAll, Korea). The target genes were amplified using Platinum Taq DNA Polymerase kit (Invitrogen, Thermo Fisher Scientific, USA) with specific forward and reverse primers (Table 3). Upon initial denaturation at 94 °C for 2 minutes, DNA was amplified in 35 cycles at 94 °C for 30 seconds, adjusted annealing temperature (depending on primer Tm) for 30 seconds, 72 °C for 45 seconds, followed by a final extension at 72 °C for 2 minutes and a cool-down. Gel electrophoresis analysis was used to determine PCR product specificity before it was purified for targeted sequencing.

**Table 3:** Oligonucleotide target primer sequences.

Target Primer	Oligonucleotide sequence (5′ → 3′)	Gene	Mutation	Amplified target region coordinates (GRCh37/hg19)
FLT3-F	5′-FAM-GCAATTTAGGTATGAAAGCCAGC	***FLT3***	bp ins in juxtamembrane domain	Chr 13: 28100592-28003274
FLT3-R	CTTTCAGCATTTTGACGGCAAC			
NPM1-F	GATGTCTATGAAGTGTTGTGGTT	***NPM1***	4 bp ins at codon 863	Chr 5: 170837476-170837653
NPM1-R	GGACAGCCAGATATCAACTG			
DNMT3A-F	TTTTCTCCCCCAGGGTATTT	***DNMT3A***	R882	Chr 2: 25457181-25457302
DNMT3A-R	GAAGAGGTGGCGGATGACT			
IDH1 132-F	GAAATATTCTGGGTGGCACG	***IDH1***	R132	Chr 2: 209113025-209113208
IDH1 132-R	CAAGTTGGAAATTTCTGGGC			
IDH2 140-F	TCTGTCCTCACAGAGTTCAAGC	***IDH2***	R140	Chr 15: 90631879-90631992
IDH2 140-R	TGGGATGTTTTTGCAGATGA			
IDH2 172-F	TCATCTGCAAAAACATCCCA		R172	Chr 15: 90631790-90631898
IDH2 172-R	CAGTGGATCCCCTCTCCAC			

### GeneScan analysis of *FLT3-ITD* mutation

Identification of *FLT3-ITD* mutant allele in sorted AML blast samples was performed by PCR with specific oligos for exon 11, labeled with 5-FAM, and with a reverse primer for exon 12 (Table 2). The reaction was conducted with: 20 ng of DNA, dNTPs mix (5 mM each), 0.1 μL SuperTherm DNA polymerase (Roche). The PCR program included 2.5 minutes at 94 °C followed by 30 cycles of 30 seconds at 94 °C, 1 minute at 57 °C, 2 minutes at 72 °C, ending with 45 seconds at 60 °C. PCR products were prepared for GeneScan reaction by addition of 9.5 μL of super DI formamide and 0.5 μL of red DNA size standard (MCLab, NimaGen, the Netherlands) in ABIP-3130 Genetic Analyzer (Applied Biosystems, Foster City, CA). Products were quantitatively analyzed using GeneMapper v4.0 software. A single pick of 328 bp determined the wild type (w.t., *FLT3*^wt^) allele, whereas the mutant allele (*FLT3-ITD*) appeared as an additional pick or as a longer product. The ITD AR was calculated by dividing the peak height of the ITD product by that of the normal w.t. product in each subpopulation.

### Targeted gene sequencing using PGM Ion Torrent

Amplicons sequencing was performed using Ion Torrent™ Personal Genome Machine® (PGM) System (Life Technologies, USA). The amplicons were quantified, pooled to generate a working library and subjected to sequencing on an Ion 314 Chip (Life Technologies). About 0.7 million reads were generated with x500 coverage per a target region. Sequences were aligned against hg19 reference genome. Variants were identified using IT Variant Caller plugin software version 4.0.37 and IGV was used to visualize the read alignment. Allelic frequency of *IDH1/2*, *NPM1* and *DNMT3A* mutations in CD34^+/-^ subpopulations was measured.

### Statistical analysis

Results were presented as mean ± SD of at least 3 independent experiments. Statistical significance was determined using two-tailed t-distribution test or ANOVA test. p < 0.05 was considered statistically significant.

### Ethics approval and consent to participate

The study was approved by the Institutional Review Board of the Rambam Health Care Campus (Approval #573-10). Informed consent was obtained from all patients before they were included in the study.

## Supplementary information

Supplementary information.

## Data Availability

The datasets used and/or analyzed during the current study are available from the corresponding author on reasonable request.

## References

[1] Ding L (2012). Clonal evolution in relapsed acute myeloid leukaemia revealed by whole-genome sequencing. Nature.

[2] Grimwade, D. & Mrozek, K. Diagnostic and prognostic value of cytogenetics in acute myeloid leukemia. *Hematol Oncol Clin North Am* **25**, 1135–1161, vii, 10.1016/j.hoc.2011.09.018 (2011). 10.1016/j.hoc.2011.09.018 22093581

[3] Grove CS, Vassiliou GS (2014). Acute myeloid leukaemia: a paradigm for the clonal evolution of cancer?. Dis. Model. Mech..

[4] Klco JM (2014). Functional heterogeneity of genetically defined subclones in acute myeloid leukemia. Cancer Cell.

[5] Gale RE (2015). Simpson’s Paradox and the Impact of Different DNMT3A Mutations on Outcome in Younger Adults With Acute Myeloid Leukemia. J. Clin. Oncol..

[6] Gaidzik VI (2013). Clinical impact of DNMT3A mutations in younger adult patients with acute myeloid leukemia: results of the AML Study Group (AMLSG). Blood.

[7] Guryanova OA (2016). DNMT3A mutations promote anthracycline resistance in acute myeloid leukemia via impaired nucleosome remodeling. Nat. Med..

[8] Lapidot T (1994). A cell initiating human acute myeloid leukaemia after transplantation into SCID mice. Nature.

[9] Bonnet D, Dick JE (1997). Human acute myeloid leukemia is organized as a hierarchy that originates from a primitive hematopoietic cell. Nat. Med..

[10] Chang, H. *et al*. Prognostic relevance of immunophenotyping in 379 patients with acute myeloid leukemia. *Leuk Res* 28, 43−48, S0145212603001802 (2004). 10.1016/s0145-2126(03)00180-2 14630079

[11] Chang H, Yeung J, Brandwein J, Yi QL (2007). CD7 expression predicts poor disease free survival and post-remission survival in patients with acute myeloid leukemia and normal karyotype. Leuk. Res..

[12] Gonen, M. *et al*. CD25 expression status improves prognostic risk classification in AML independent of established biomarkers: ECOG phase 3 trial, E1900. *Blood* **120**, 2297–2306, 10.1182/blood-2012-02-414425 (2012). 10.1182/blood-2012-02-414425 PMC3447784 22855599

[13] Cerny J (2013). Expression of CD25 independently predicts early treatment failure of acute myeloid leukaemia (AML). Br. J. Haematol..

[14] Raspadori D (2001). CD56 antigenic expression in acute myeloid leukemia identifies patients with poor clinical prognosis. Leukemia.

[15] Zaidi SZ, Motabi IH, Al-Shanqeeti A (2016). CD56 and RUNX1 isoforms in AML prognosis and their therapeutic potential. Hematol. Oncol. Stem Cell Ther..

[16] Xu, S., Li, X., Zhang, J. & Chen, J. Prognostic value of CD56 in patients with acute myeloid 10.1007/s00432-015-1977-3 (2015). 10.1007/s00432-015-1977-3 PMC11823892 25924702

[17] Bachas C (2012). The role of minor subpopulations within the leukemic blast compartment of AML patients at initial diagnosis in the development of relapse. Leukemia.

[18] de Boer B (2018). Prospective Isolation and Characterization of Genetically and Functionally Distinct AML Subclones. Cancer Cell.

[19] Wang L (2013). FISH+CD34+CD38- cells detected in newly diagnosed acute myeloid leukemia patients can predict the clinical outcome. J. Hematol. Oncol..

[20] Schuurhuis GJ (2013). Normal hematopoietic stem cells within the AML bone marrow have a distinct and higher ALDH activity level than co-existing leukemic stem cells. PLoS One.

[21] Ofran Y, Rowe JM (2011). Induction and postremission strategies in acute myeloid leukemia: what is new?. Curr. Opin. Hematol..

[22] Asou H (1991). Establishment of a human acute myeloid leukemia cell line (Kasumi-1) with 8;21 chromosome translocation. Blood.

[23] Pedranzini, L. *et al*. Differential cytogenomics and miRNA signature of the Acute Myeloid Leukaemia Kasumi-1 cell line CD34+38- compartment. *Leuk Res* **34**, 1287–1295, S0145- 10.1016/j.leukres.2010.02.012 (2010). 10.1016/j.leukres.2010.02.012 20227111

[24] Hsu, C. L., Kikuchi, K. & Kondo, M. Activation of mitogen-activated protein kinase kinase (MEK)/extracellular signal regulated kinase (ERK) signaling pathway is involved in myeloid lineage commitment. Blood 110, 1420-1428, blood-2007-02-071761 (2007). 10.1182/blood-2007-02-071761 PMC1975832 17536016

[25] Chan G, Gu S, Neel BG (2013). Erk1 and Erk2 are required for maintenance of hematopoietic stem cells and adult hematopoiesis. Blood.

[26] Staser K (2013). Normal hematopoiesis and neurofibromin-deficient myeloproliferative disease require Erk. J. Clin. Invest..

[27] Geest CR, Coffer PJ (2009). MAPK signaling pathways in the regulation of hematopoiesis. J. Leukoc. Biol..

[28] van Stijn A (2003). Differences between the CD34+ and CD34- blast compartments in apoptosis resistance in acute myeloid leukemia. Haematologica.

[29] Milella M (2001). Therapeutic targeting of the MEK/MAPK signal transduction module in acute myeloid leukemia. J. Clin. Invest..

[30] Nishioka C, Ikezoe T, Yang J, Yokoyama A (2009). Inhibition of MEK signaling enhances the ability of cytarabine to induce growth arrest and apoptosis of acute myelogenous leukemia cells. Apoptosis.

[31] Li P (2016). Inhibition of Mnk enhances apoptotic activity of cytarabine in acute myeloid leukemia cells. Oncotarget.

[32] Casado, P. *et al*. Kinase-substrate enrichment analysis provides insights into the heterogeneity of signaling pathway activation in leukemia cells. *Sci Signal* **6**, rs6, 10.1126/scisignal.2003573 (2013). 10.1126/scisignal.2003573 23532336

