# Supplementary material for: ERK Activity in Immature Leukemic Cells Drives Clonal Selection during Induction Therapy for Acute Myeloid Leukemia
Source: Sci Rep. 2020 May 20;10:8349. doi: 10.1038/s41598-020-65061-6 (PMC7239856; doi:10.1038/s41598-020-65061-6)

ERK Activity in Immature Leukemic Cells Drives Clonal Selection during Induction Therapy for Acute Myeloid Leukemia

Michal Hayun[1], Maria Zaatra[1,2], Chen Itzkovich[1,2], Dvora Sahar[3], Dina Rosenberg[3], Margarita Filatova[3], Shimrit Ringelstein-Harlev[3,4], Hagit Baris[1, 5], Nivin Moustafa-Hawash[5], Igal Louria-Hayon[1,] and Yishai Ofran[1,2,4]

Supplementary Fig.1

Example (of one patient) for gating strategy for CD34$^{+/-}$ selection in specimens derived from 60 AML patients

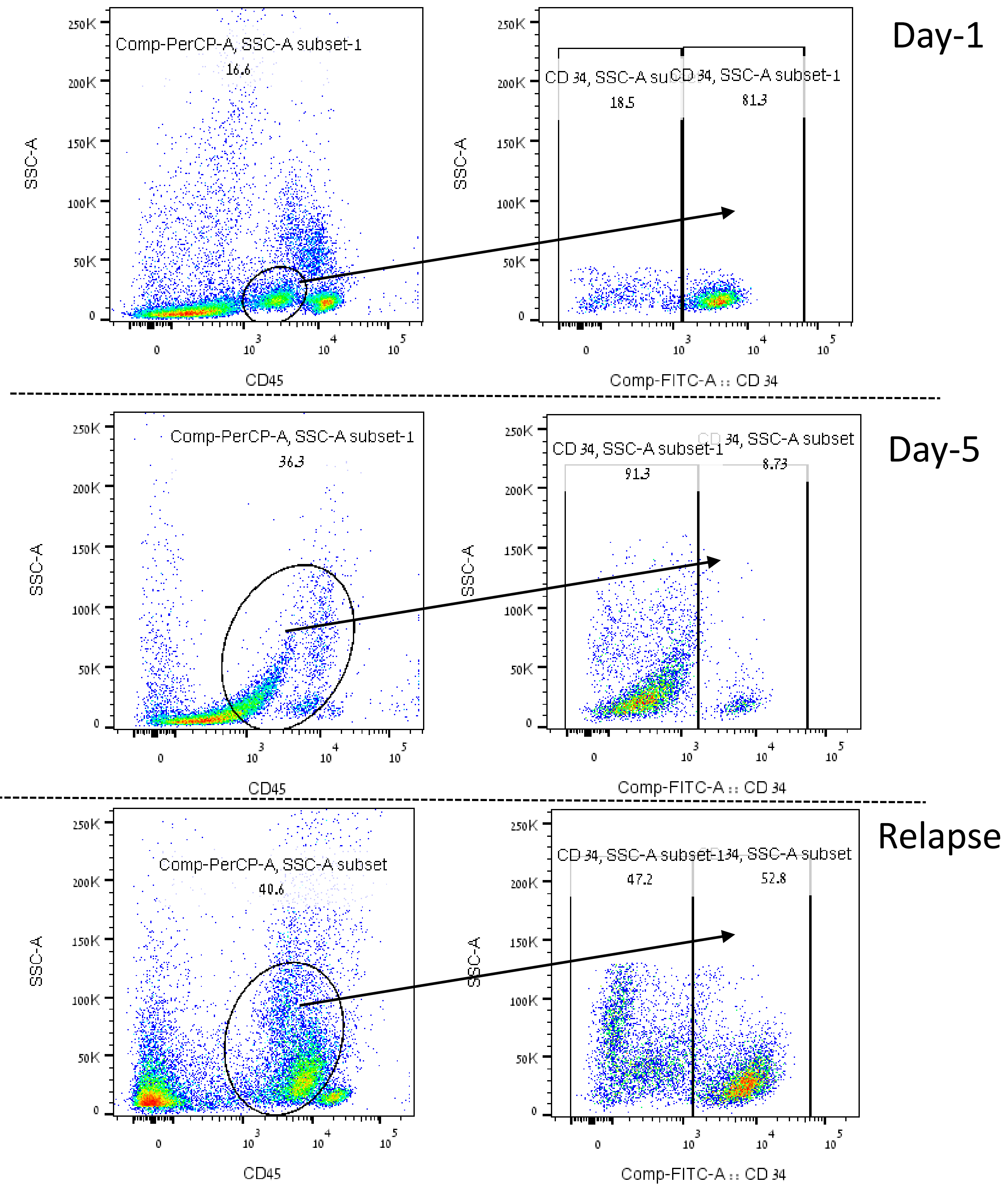

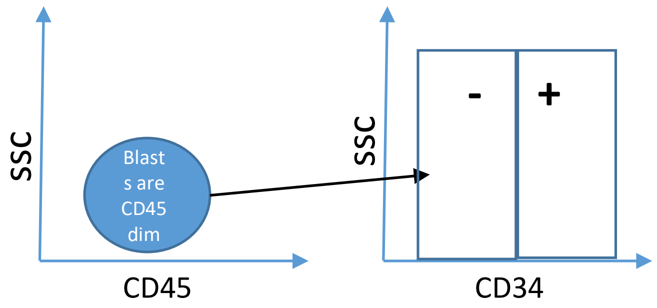

Supplementary Information to figure 2A

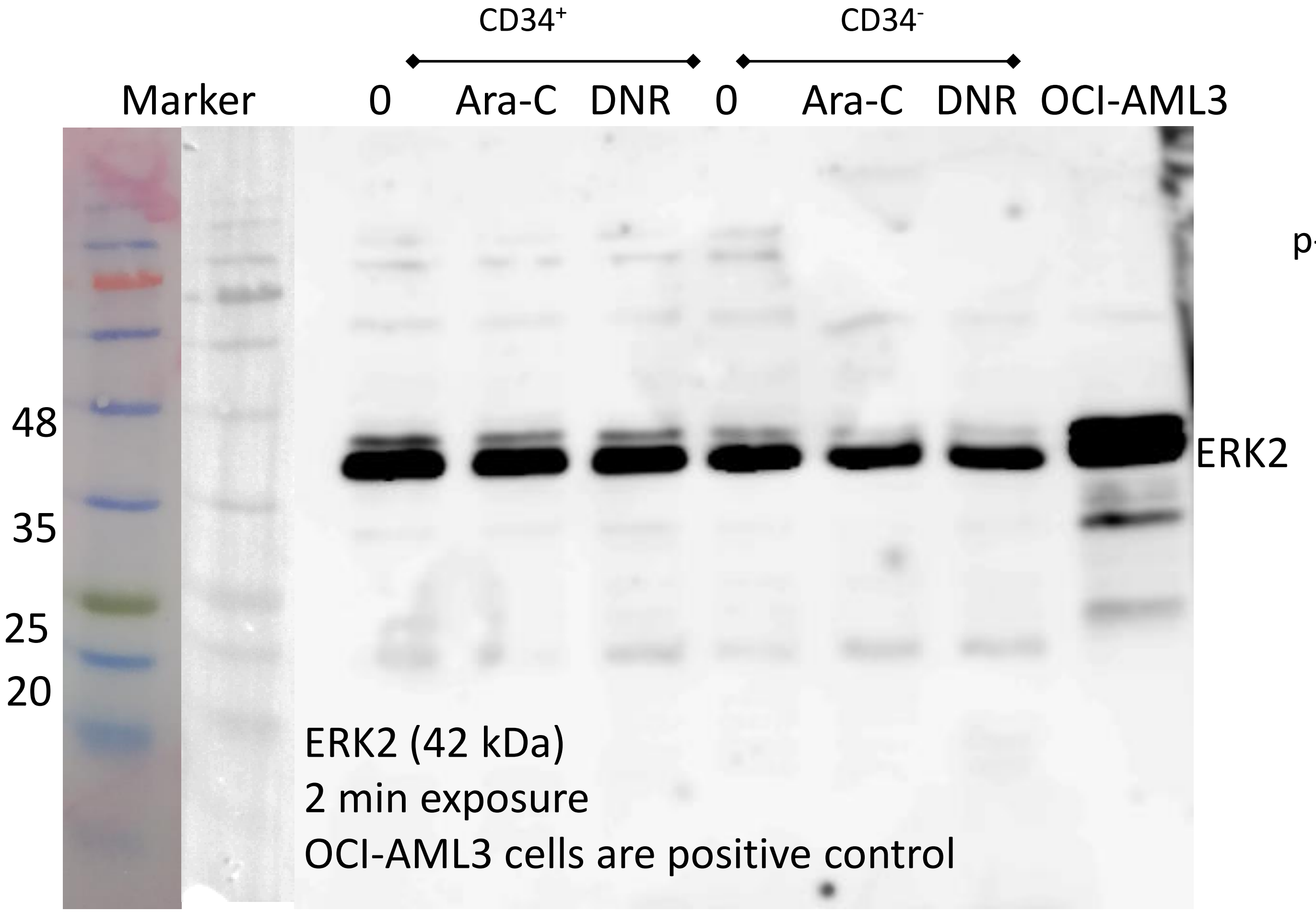

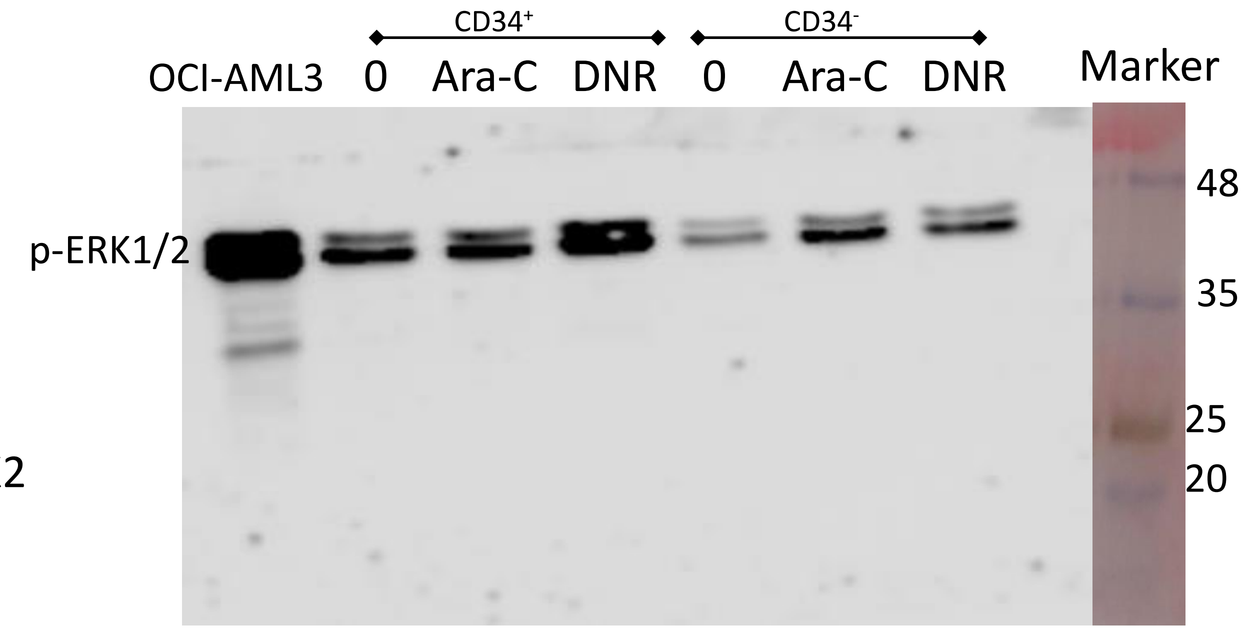

p-ERK1/2 (44/42 kDa)
10 min exposure
OCI-AML3 cells are positive control

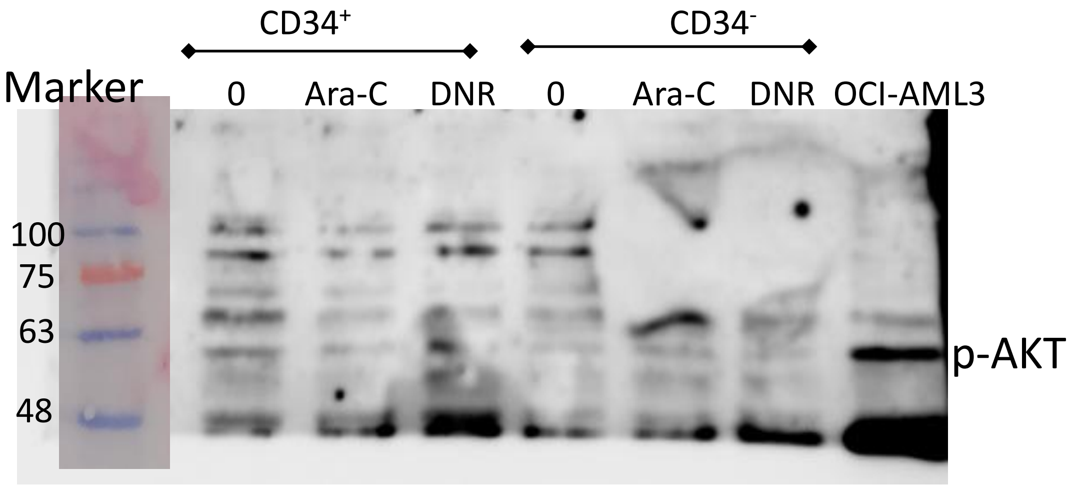

p-AKT
(60 kDa) 10 min exposure
OCI-AML3 cells are positive control

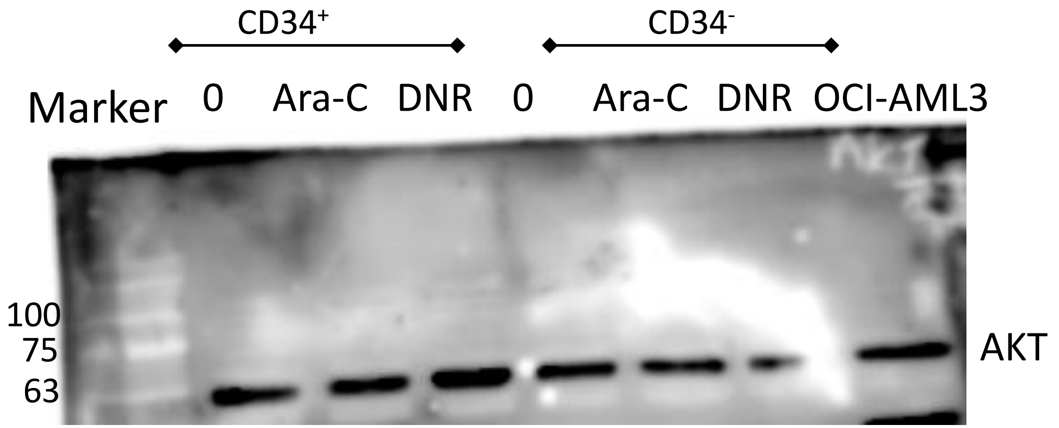

Akt (60 kDa)
2 min exposure
OCI-AML3 cells are positive control

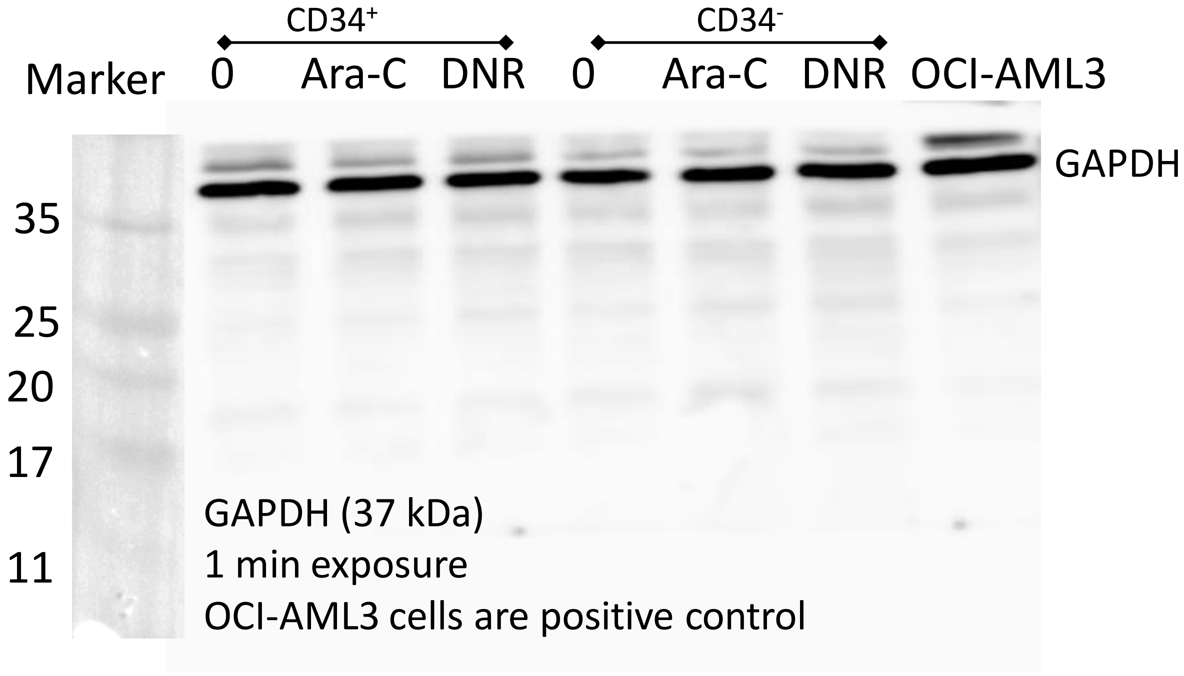

GAPDH (37 kDa)
1 min exposure
OCI-AML3 cells are positive control

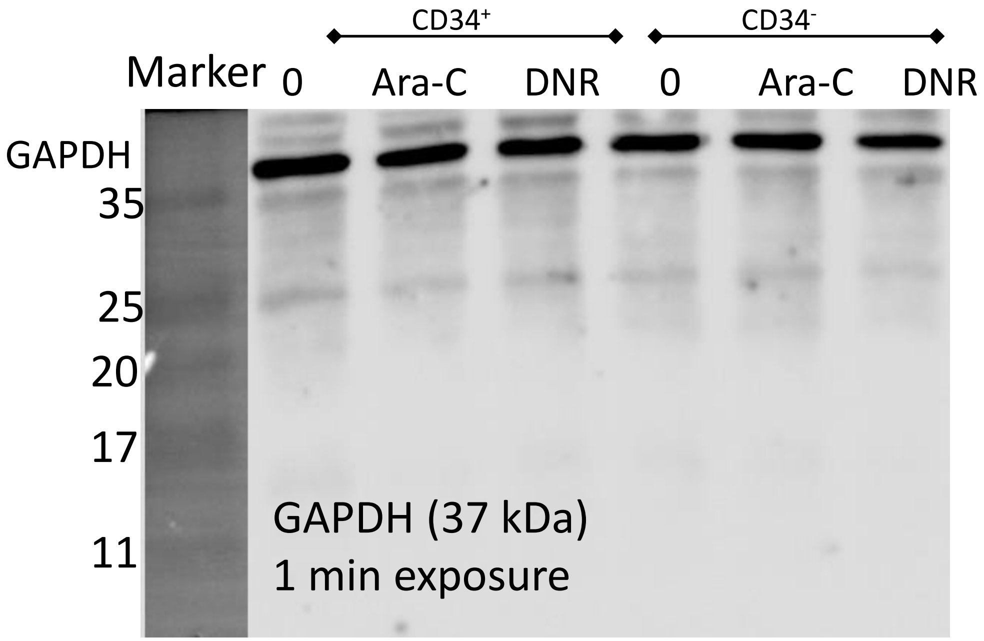
CD34⁺
CD34⁻
Marker
0
Ara-C
DNR
0
Ara-C
DNR
GAPDH
35
25
20
17
11
GAPDH (37 kDa)
1 min exposure

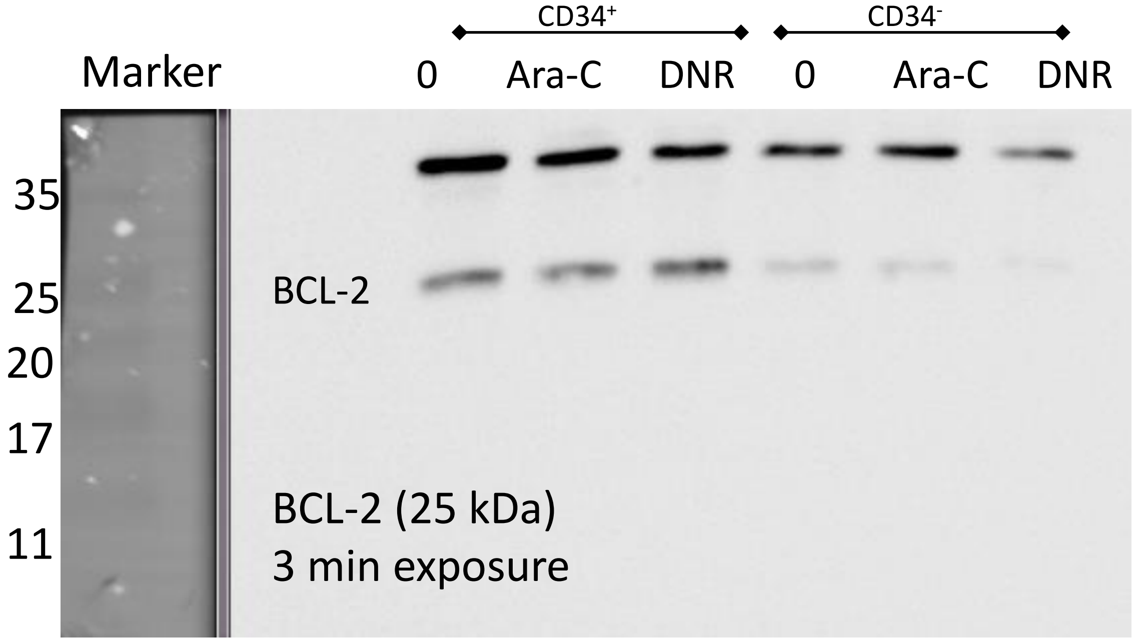
CD34⁺
CD34⁻
Marker
0
Ara-C
DNR
0
Ara-C
DNR
35
25
20
17
11
BCL-2
BCL-2 (25 kDa)
3 min exposure

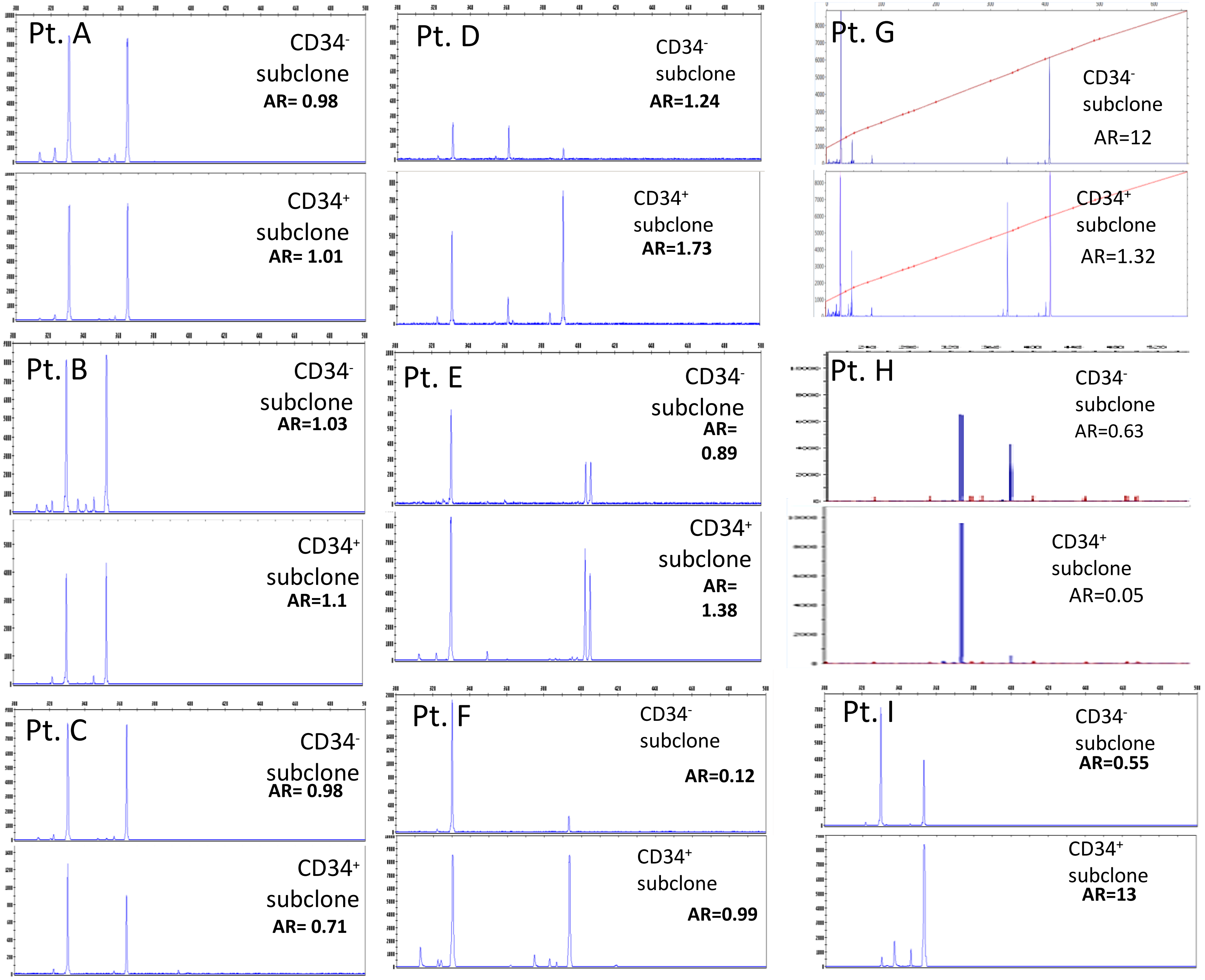

Supplementary Fig.2

Example (Pt. A)
for gating strategy of $CD34^{+/-}$ subsets
sorted from FLT3-ITD patients.

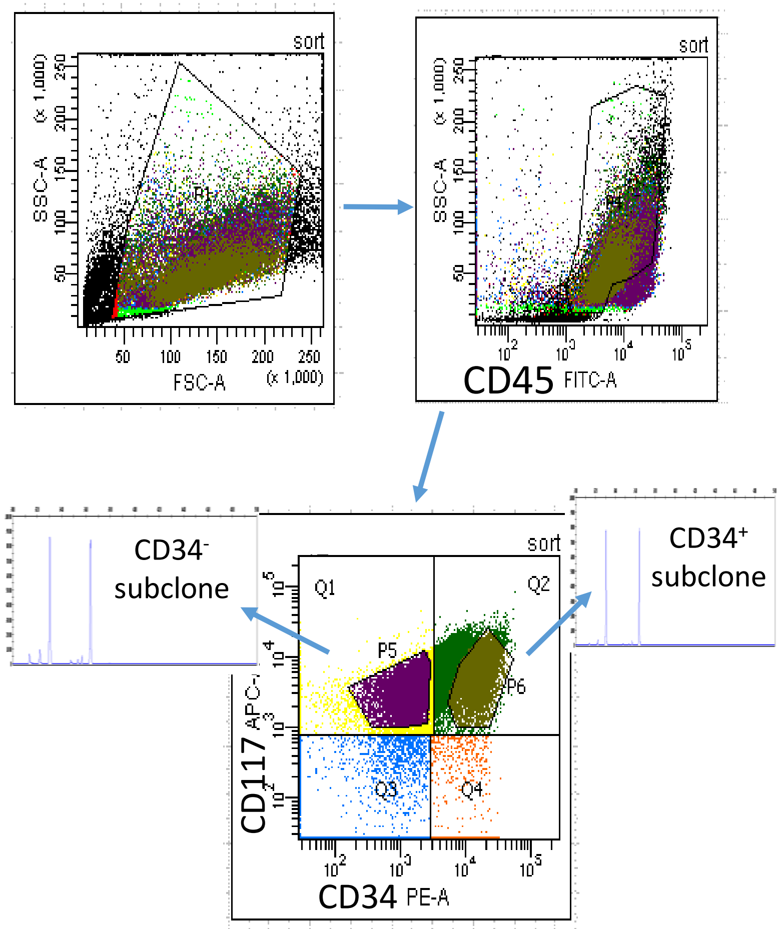

Supplement: Supplementary file 1 — Supplementary information. [file 41598_2020_65061_MOESM1_ESM.pdf]
